# Modulation of Visually Evoked Postural Responses by Contextual Visual, Haptic and Auditory Information: A ‘Virtual Reality Check’

**DOI:** 10.1371/journal.pone.0067651

**Published:** 2013-06-28

**Authors:** Georg F. Meyer, Fei Shao, Mark D. White, Carl Hopkins, Antony J. Robotham

**Affiliations:** 1 Department of Experimental Psychology, Liverpool University, Liverpool, United Kingdom; 2 The Virtual Engineering Centre, STFC Daresbury Laboratory, Daresbury Science and Innovation Campus, Warrington, United Kingdom; 3 Flight Science & Technology, Department of Engineering, University of Liverpool, Liverpool, United Kingdom; 4 Acoustics Research Unit, School of Architecture, University of Liverpool, Liverpool, United Kingdom; 5 School of Engineering, Auckland University of Technology, Auckland, New Zealand; ICREA-University of Barcelona, Spain

## Abstract

Externally generated visual motion signals can cause the illusion of self-motion in space (vection) and corresponding visually evoked postural responses (VEPR). These VEPRs are not simple responses to optokinetic stimulation, but are modulated by the configuration of the environment. The aim of this paper is to explore what factors modulate VEPRs in a high quality virtual reality (VR) environment where real and virtual foreground objects served as static visual, auditory and haptic reference points. Data from four experiments on visually evoked postural responses show that: 1) visually evoked postural sway in the lateral direction is modulated by the presence of static anchor points that can be haptic, visual and auditory reference signals; 2) real objects and their matching virtual reality representations as visual anchors have different effects on postural sway; 3) visual motion in the anterior-posterior plane induces robust postural responses that are not modulated by the presence of reference signals or the reality of objects that can serve as visual anchors in the scene. We conclude that automatic postural responses for laterally moving visual stimuli are strongly influenced by the configuration and interpretation of the environment and draw on multisensory representations. Different postural responses were observed for real and virtual visual reference objects. On the basis that automatic visually evoked postural responses in high fidelity virtual environments should mimic those seen in real situations we propose to use the observed effect as a robust objective test for presence and fidelity in VR.

## Introduction

Immersive virtual environments are increasingly used for visualization, in training, rehearsal, rehabilitation and in many other areas (review [1]). There is a strong argument that by increasing immersion (the objective level of sensory fidelity that a VR system provides [2]) task performance is improved [3], [4].

The illusion of self-motion in space (vection) can be very compelling and can make a major positive contribution to the overall experience and effectiveness of VR systems [5], [6]. Unfortunately, vection may also be perceived where it is not intended, for example when object motion of large-scale visual 3D models is unintentionally perceived as self-motion. Motion signals leading to vection can also cause mild to severe discomfort, such as motion sickness or motion after-effects, which some users experience during or after exposure to VR environments [7]–[9].

The factors modulating vection and immersion in VR environments have the potential to provide an effective means to optimise VR environments to either enhance or suppress self-motion illusions. Therefore the benefits of providing compelling signals that lead to vection have to be considered in the context of potentially undesirable side-effects.

Visual motion signals that lead to vection also contribute to automatic postural responses that are necessary to maintain upright stance during self-motion. Human postural behaviour is strongly dependent upon input from the visual [10], somatosensory [11] and vestibular systems [12]–[14]. Visual information, including peripheral motion perception [15], provides the dominant input to postural stability during quiet, unperturbed stance, because of the relatively high sensitivity of the visual motion detection system compared with the proprioceptive and vestibular inputs. Balance is maintained when vision is denied, but body sway typically doubles in amplitude [16].

Visual signals that cause vection also cause visually evoked postural responses (VEPR) that are coupled dynamically to the visual motion [17] and modulated by the environment to generate appropriate responses for the visual signals seen [18]. Vection increases the magnitude and accuracy of visually evoked postural responses [19], consistent with the maintenance of a stable balance during self-motion [10], [16], [20], [21]. The magnitude of these adjustments is influenced by stimulus characteristics, such as stimulus area, velocity and spatial frequency [22], [23] and environmental factors, such as the stability of the support base that participants stand on [24], the point of fixation [18] and additional positional cues that can be represented in all sensory modalities [12], [25].

Guerraz and Bronstein [26] argue that, whilst vection and VEPR draw on common representations, they are not causally related because VEPRs, in contrast to vection, are initiated by transient visual stimuli, which can be measured well before vection is perceived and are modulated by the geometry of the visual display. They argue that VEPRs are controlled by a short latency, automatic and subconscious system, which also controls natural body sway; whilst vection contributes to a second, much longer latency system, which draws on cortical pathways and contributes to postural control during prolonged body displacements.

The aim of this study is to explore how environmental factors modulate visually evoked postural sway, in particular the transient VEPR component.

### Modulation of Postural Sway by Environmental Factors

**Figure 1:**
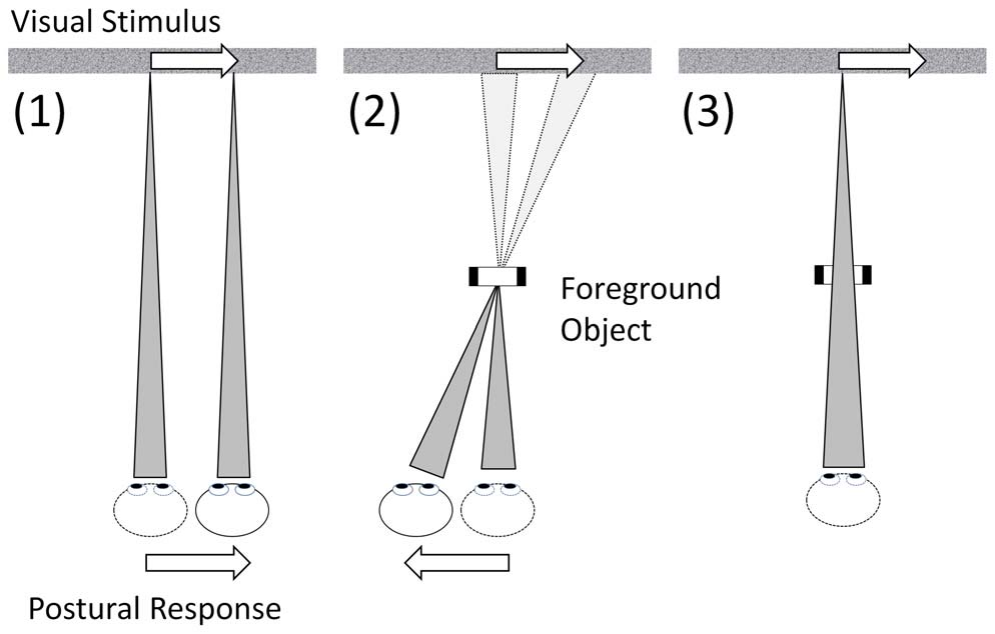
Schematic diagram of the three experimental conditions from Bronstein and Buckwell [18] used as the basis for our experiments. Postural responses in response to the same visual translating stimulus were measured in three conditions. When participants fixate a moving background postural responses follow the background motion (1), when the moving background is fixated through a static window (2), the postural response is explained by postural control driven by parallax cues. When participants fixate the foreground object (3) no visually evoked postural sway is seen.

Bronstein and Buckwell [18] provided first evidence that postural reactions elicited by lateral visual motion are *not* rigid responses to optokinetic stimulation but are modulated by the configuration of the environment. Their subjects viewed a visual background display, which moved laterally for 2s under the following three conditions: (1) direct fixation of the background, (2) fixation of a stationary object (a window frame) in the foreground, and (3), fixation of the background through the foreground, see Fig. 1. Participants moved with the stimulus in condition (1), but swayed in the opposite direction when fixating the foreground object while the background moved in condition (2). This behaviour is appropriate for maintaining a stable stance during (perceived) self-motion in parallel with the screen, while the observed posture change in the opposite direction in condition (2) is appropriate when parallax motion (pivoting around the fixation point) is perceived. When participants fixate the background through a static foreground window, condition (3), no visually-evoked postural sway is observed. Bronstein and Buckwell [18] argue that in this condition the visual stimulus is not congruent with what would be seen for self motion: one would expect to perceive motion of the background and, to a lesser degree, of the foreground.

Bronstein and Buckwell [18] argue that postural control relies on automatic multi-sensory processing and motor responses of which we are largely unaware. Since the visually evoked postural responses are modulated by the configuration of the environment and fixation point, the control of sway cannot result from a rigid optokinetic reflex to simple motion stimuli, but instead must draw on a range of visual motion cues, including congruent visual parallax cues, and a high level scene interpretation. The data shows that a static visual reference point can significantly reduce postural sway. The results were replicated by Guerraz and colleagues [26], [27] who showed that the behavioural data is consistent with the two different postural control mechanisms that act with different time constants and draw on different representations: a short latency system that is driven by transient visual stimuli, including parallax cues, and a longer latency, vection enhanced, postural control system that draws on consciously perceived self motion.

### Multisensory Interactions

Behavioural data show that temporally, spatially, and semantically congruent information represented in more than one modality facilitates performance: for example, audio-visual congruent motion stimuli are detected and discriminated faster and more accurately than unimodal or incongruent bimodal stimuli [28]–[30]. The facilitatory effect of spatial and temporal congruence can be explained by early neural integration stages that have, for instance, been demonstrated in the superior colliculus of cat [31], [32], but multisensory interactions are also seen when signals in more than one modality present the same semantics [33]. Other modalities, such as proprioception or hearing, therefore contribute additional contextual information that may modulate visually evoked postural sway.

Somatosensory signals are a key component in posture maintenance and can modulate visually evoked postural sway responses. Peterka and Benolken [12] characterised the influences of vestibular and somatosensory cues to postural sway in normal and bilateral vestibular absent subjects and found that in both subject groups visually evoked postural sway was significantly reduced when somatosensory cues provided orientation information. A simple manipulation to significantly reduce visually evoked postural sway is to ask participants to lightly touch a static surface with a finger [34], [35].

Static audio signals have also been shown to reduce natural postural sway. Easton, Green, DiZio and Lackner [25], for example, showed that 500 Hz square waves at 73 dB(A) emitted by two loudspeakers placed adjacent to each ear significantly reduce natural postural sway for blind and sighted participants. Auditory cues have been used to facilitate posture regulation by providing a spatial anchor to elderly participants [36], or as audio biofeedback devices that reduce postural sway in patients with vestibular loss [37], [38].

### Summary of Experimental Aims

Experiment 1 of this study was designed to extend original work by Bronstein and colleagues [18], [26] who showed that the presence of a static visual reference modulates transient automatic VEPRs.

The experiments extend the original studies by:

1. Presenting a virtual visual reference, but also tactile and auditory static anchor points.
2. Presenting medial-lateral motion stimuli as well as anterior-posterior visual motion signals,
3. Presenting a repeating stimulus of 3×0.5 Hz sinusoidal movements rather than a single cycle of motion to further test the temporal interactions of the putative competing mechanisms [26].

The data obtained in experiment 1, using a virtual visual anchor differ significantly from what we expected to see on the basis of previous data that used real foreground objects [18], [27]. We hypothesize that these differences are result of the virtual presentation of the foreground object in our experiments compared to the physical foreground object used in the original study. In a second set of experiments, we therefore directly compare visually evoked postural responses for matching virtual and real foreground objects.

## Methods

### Ethics Statement

The experiments have been approved by the University of Liverpool ethics committee (reference PSYC-1112–049). Written informed consent was acquired from all participants.

### Overview

We describe four experiments, conducted with two different cohorts of ten participants, which share a common experimental design and procedures. In all experiments visually evoked postural responses to motion of a background pattern in a virtual environment were measured.

In the first pair of experiments the effects of fixation (foreground/background) and the presence of visual, haptic and auditory spatial reference points is explored. In experiment 1a the background moves in a lateral direction while in experiment 1b the same conditions are probed with anterior-posterior motion.

The second set of experiments compare the effect of presenting real and virtual foreground objects and their fixation for lateral (experiment 2a) and anterior-posterior motion (experiment 2b).

### Participants

Two distinct groups of 10 participants each, recruited via opportunity sampling, took part in the experiments.

Group 1 (experiment 1a and 1b): age range 19–50 years, mean 24.2 years; six males.

Group 2 (experiment 2a and 2b): age range 18–46 years, mean 24.9 years; three males.

All participants reported normal or corrected-to-normal vision and normal hearing. None had a history of vestibular disorder.

### VR Set-up

The installation of a VR system in the laboratories of the Virtual Engineering Centre provided the opportunity to reproduce the experiments of Bronstein and Buckwell [18], [26] in a virtual world. The laboratory consists of a planar display screen of length 6.0 m and height 2.1 m behind which are two active stereo projectors that create 3390 x 1200 resolution images at a rate of 120 Hz. Observers wear wireless LCD shutter glasses that are synchronized with the projectors to provide stereoscopic images. The position of the shutter glasses is tracked using 12 VICON Bonita infrared cameras at a resolution<±0.1 mm. Position data, computed using VICON Tracker software, is broadcast in real-time across the internal network using a VRPN protocol at a rate of 200 Hz and used to update the virtual environment. The position of the 3D-glasses in space was recorded to provide the head position data used in the analysis of postural sway.

Immersive virtual reality experiences are created using the 3DVIA Virtools application, which integrates 3D virtual environments to the display technology, tracking system and interactive devices [39].

**Figure 2:**
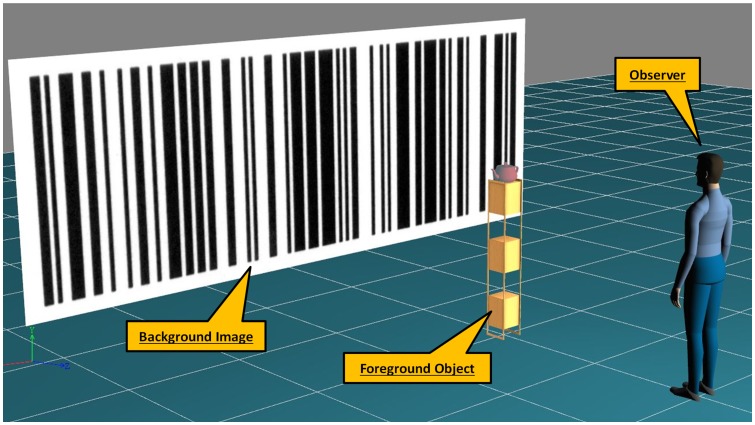
Elements of the Virtual Reality Environment used in all experiments: the translating background image (a barcode), the foreground object (a virtual or real teapot on a stand) and an avatar representing the observer in the scene.

The virtual reality environment (Fig. 2) consists of three major elements:

The background image: an image of a barcode extending across the 6 m×2.1 m display screen, at a distance of 4 m in front of the observer (86° visual angle horizontally).

**Figure 3:**
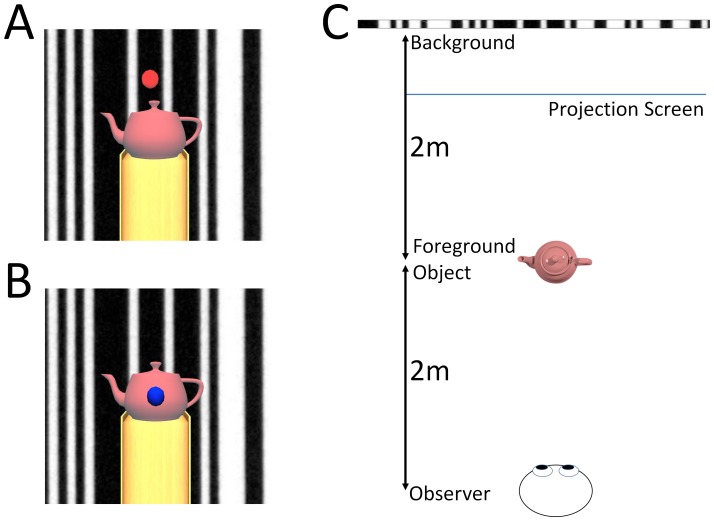
The stimuli from the viewer’s perspective. Participants were asked to press a button when the fixation target (either a red dot on the background image (A) or a blue dot on the foreground object (B)) transiently disappeared. Panel C shows a plan view of the room layout: Participants viewed a moving image of a barcode 4 m ahead of them. A real or virtual teapot on a stand 2 m from the observer was used as a foreground object.

The foreground object: a 3D geometric model of a teapot sitting on top of a stand, 2 m away from the observer subtending 4.6° visual angle (Fig. 3).

The observer: a virtual camera whose position and orientation are directly linked to that of the real-life observer through the tracking system to dynamically render images on the display screen that will appear to the observer as a true perspective of the scene [39]. The avatar shown in Fig. 2 is not visible.

The room was darkened during all experiments. Participants stood with their feet parallel to each other and approximately 20 cm apart on a 50 mm foam pad to reduce the accuracy of lower limb proprioceptive signals so enhancing the influence of vision on sway control [40], [41].

In conditions where an audio signal was presented as a reference signal, white noise was presented at 50 dB (A), measured in the participant’s ear position, using a loudspeaker, positioned 60 cm to the right of the participant at a height of 141 cm. White noise was chosen to maximise sound localisation performance [42].

In the condition where a tactile reference was given, participants were asked to lightly place the tip of their right index finger on a stand at a height of 102 cm, 30 cm in front and to the right of the observer. The position provides reliable tactile cues but not mechanical support.

Participants were asked to fixate a coloured point, either on the background (approximately 10° vis. angle above the foreground object) or on the foreground object (Fig. 3). In conditions where the real teapot was used as a visual anchor, a laser pointer, directed at the pot, was used as the fixation target. The object was not specifically illuminated, but the glare from the projection screen behind it made it easy to see.

### Stimuli

The virtual environment was manipulated in two experimental blocks containing either medio-lateral or anterior-posterior visual motion of the background image, an image of a barcode to provide a rich motion stimulus. The order of presentation was counterbalanced across the participant pool. Each block lasted 20 minutes and participants had a minimum of 20 minutes rest between the two successive blocks.

Within each block five conditions were run that consisted of 20 presentations of a 6 s visual motion pattern that preceded 6 s of a static display (rest condition) during which natural postural sway was measured.

The dynamic characteristics of the visual stimulus follows [18] and was chosen to be within the range of those present in spontaneous body sway, consistent with the aim to present motion signals that would be interpreted as being caused self-motion. The motion signals consisted of three raised-cosine oscillations at a frequency of 0.5 Hz and maximum amplitude of 50 mm (0.7° visual angle in the lateral motion condition) relative to the origin.

In the lateral sway condition alternating movements from the origin to the left or right and back were presented while in the anterior-posterior condition the motion was always from the origin toward the observer and back again.

### Task

Participants were asked to stand as still as possible, look at a fixation target (Fig. 3) and press a button when the target transiently disappeared. Responses were elicited at random intervals (approximately 1 event every 5 s, and 1 s minimum gap between events) throughout the entire block, which contained equal periods of moving and static displays.

### Data Analysis

Head position data was recorded using a VICON motion tracking system by tracking IR reflective markers on the 3D shutter glasses worn by the participants.

Upright balance is maintained via low-frequency lower-limb muscle activity (2.5 Hz), which is mechanically filtered to produce an even lower frequency body sway (<1 Hz, review Dakin et al. [43]). During natural postural sway, more than 90% of motion energy in the anterior-posterior direction and more than 95% of motion energy in the lateral direction is observed below 2 Hz [44], [45]. To account for this the recorded data was filtered off-line using a second order zero-lag Butterworth band-pass filter with cut-off frequencies of 0.125 Hz and 2.5 Hz and sampled at 10 Hz. The standard deviation of the filtered motion signals in X (lateral) and Z (anterior-posterior) direction was then computed for individual trials to quantify the total motion within each trial and trials with a standard deviation greater than three times the mean standard deviation within each block were discarded to prevent abnormal motion patterns from distorting the mean data. In total 7% of trials with lateral motion and 4% of trials in the anterior-posterior motion direction were discarded.

**Figure 4:**
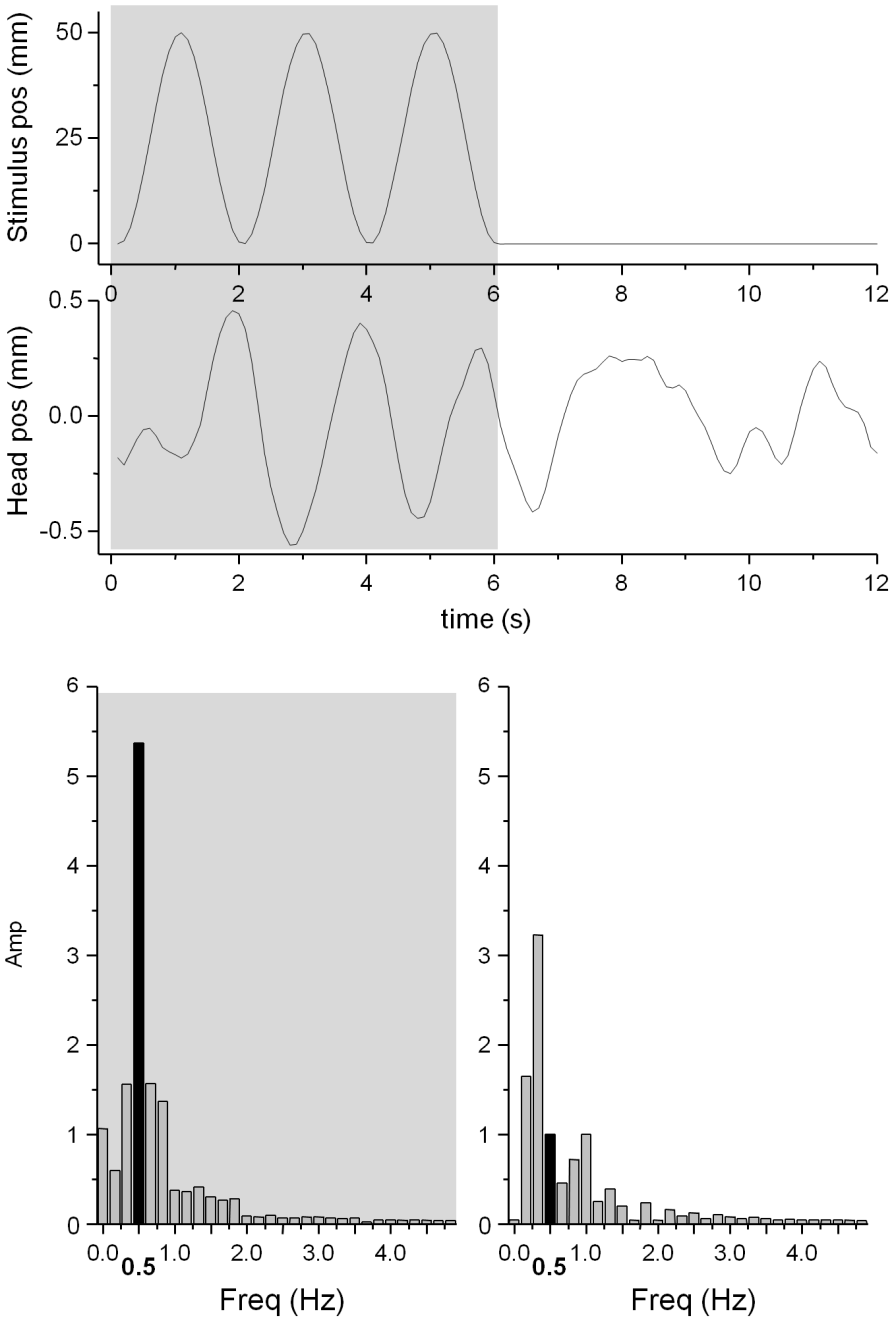
Sample Stimulus and response pair (Fixate background only exp. 2) to show the stimulus motion in a lateral direction (top) and the average head sway as a result. The visual evoked stimuli were presented for three cycles/six seconds at 0.5 Hz (grey underlay), followed by a six second pause where a static visual scene was displayed. To quantify visually evoked motion the proportion of energy of the head motion at the stimulus frequency of 0.5 Hz (black bar in the lower two figures) relative to the total energy in the spectrum was computed. During the initial six seconds a large spectral component (30% of the total energy) at the stimulus frequency is clearly visible while during the final six seconds energy at 0.5 Hz accounts for only 10% of the total energy.

VEPRs are dynamically coupled to the stimulus [17]. To quantify visually evoked postural responses for our sinusoidal stimuli, the proportion of energy of head motion at the stimulus frequency (0.5 Hz) relative to the total energy in the spectrum was computed (Fig. 4). If the head position precisely tracks the visual stimulus motion, 100% of the spectral energy would be at 0.5 Hz. When no visual stimulus is present and natural body sway is observed, a wide spectral distribution around 1 Hz is expected [46], Fig. 4.
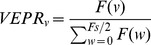
(1)Where VEPR is the amplitude of the Fourier component, *F(v),* at the visual stimulus frequency, *v = 0.5 Hz*, relative to the total energy in the spectrum between 0 Hz and half the sampling frequency (*F_s_/2 = 5 Hz*) *(* *eqn. 1* *)*. To quantify visually evoked postural responses we compare this measure for the 6 s stimulus presentations with that for the 6 s pause immediately following the stimulus. This ensures that individual differences or sequence effects are controlled for. The signal measured during visual stimulation contains natural sway as well as an evoked response. Averaging the measured response reduces this natural sway component because only the signal component is phase locked into the stimulus. This reduction in noise means that the population average response provides a better estimate of the evoked signal amplitude. In the following analysis we therefore report the population average.

To estimate the mean and variance of the population responses we used standard bootstrapping (e.g. [47]) to estimate the sampling distribution by resampling the postural sway data of our participant pool. For all data reported here the means and variance estimates are based on 1000 resamples of the observed dataset, obtained by random sampling with replacement of the population data.

### Statistical Analysis

All four experiments share a similar design and analysis. Each experiment tests VEPRs in five experimental conditions. Visual background motion in lateral (experiments 1a and 2a) and fronto-posterior (experiments 1b and 2b) direction is presented and visually evoked postural responses are measured in both directions.

The analysis is conducted in two stages:

1. VEPRs should match the direction of the visual stimulation: lateral head motion is expected for laterally moving visual stimuli but not for visual stimuli that move in the anterior-posterior direction, and vice versa.
2. An ANOVA with the factors ‘experimental condition’ (5) x ‘visual stimulus motion’ (on/off, 2) and ‘subject’ as a random factor was computed for each of the two orthogonal head motion directions. Planned comparisons, as detailed below, were only carried out where significant main effects of ‘visual stimulus motion’ or interactions were evident.
3. Visual stimulus motion should cause significant increases of the VEPR relative to the pause immediately following the stimuli (our control condition). Planned comparisons, using one-tailed t-tests, are therefore used. In experiment 1 five planned comparisons, one for each experimental condition, are conducted. The five a-priori hypotheses were evaluated using Bonferroni adjusted alpha levels of.01 per test (.05/5).
4. In experiment 2, seven planned comparisons were conducted: one for each of the five experimental conditions, as in experiment one, and two additional direct comparison of the VEPR for matching real and virtual foreground conditions. Bonferroni adjusted alpha levels of 0.0071 per test (.05/7) were used for hypothesis testing. All statistical tests on these population estimates were performed using independent (two-sample) t-tests [48] because bootstrapping was used to estimate the sample population mean and variance.

## Results

### Experiment 1: The Effect of Static Multi-sensory Reference Signals on Visually Evoked Postural Responses

It is well known that *natural* postural sway can be reduced by the provision of static reference signals in other modalities, such as vision (e.g. [10]), touch [34], [49], or auditory cues [25], [50]. Our aim was to test whether virtual visual, auditory, or tactile foreground references also modulate the VEPR, in particular the transient component that was shown to lead to counter-directional postural responses when the visual signals are consistent with parallax motion [26].

Lateral and anterior-posterior motion was presented in separate, counterbalanced experiments.

Each experiment consisted of five conditions:

1) BG only - fixate a target on the background (BG), no foreground objects present.

2) BG visual - fixate a target on the background, a foreground object is present.

3) BG touch - fixate a target on the background, lightly touch a surface in front.

4) FG visual - fixate a target on the foreground (FG) object.

5) BG audio - fixate a target on the background, a static, white noise, audio signal is played.

Conditions 1, 2 and 4 are similar to those used in [18].

#### Behavioural performance

To ensure that participants attended the stimulus and to control fixation, participants were asked to press a manual response button whenever a fixation target, which could be either at eye-height on the background or one the foreground object, transiently disappeared. Participants correctly detected 97.34% (SD 6.92) of the target events during the lateral motion condition and 99.06% (SD 1.83) in the anterior-posterior direction. No participant scored below 90% in individual blocks.

#### Experiment 1a: Postural responses to laterally moving visual stimuli

Based on previous work we hypothesized that not only a visual anchor, but also tactile and auditory static reference points would reduce postural sway in response to visual background motion.

**Figure 5:**
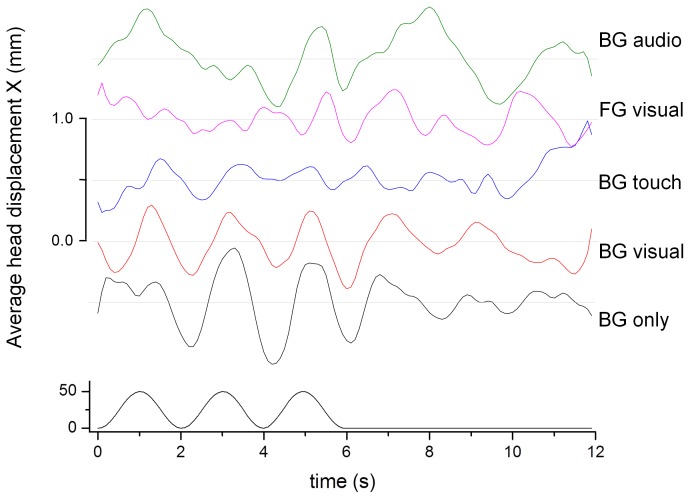
Average head displacement (top) in response to the laterally moving visual stimulus (bottom) in mm. The graphs are offset by 0.5 mm to enhance visibility. The labels identify the fixation point (BG/FG: background/foreground object fixation) and the type of anchor provided: visual – a rendered object in the foreground, touch – light finger touch, audio – a static white noise audio source.

Figure 5 shows the average head displacement for our ten participants in the five conditions for laterally moving visual stimuli. The stimulus, three cycles at 0.5 Hz with a maximum displacement of 5 cm, is shown at the bottom. The stimulus driven response is clearly visible in conditions ‘BG only’ (fixate background, no foreground present) and ‘BG visual’ (fixate background, visual foreground object present).

To quantify the VEPR, the proportion of motion energy at the visual stimulus frequency (0.5 Hz) relative to the total energy in the spectrum was computed (eqn. 1) during the stimulus condition and is compared to the magnitude of natural postural sway at this frequency during rest. The analysis windows in both cases extended over the full 6 s of stimulus or rest.

A repeated measures ANOVA with the factors ‘condition’, ‘visual motion’ and ‘subject’ as random factor showed a significant main effect of visual motion (F(1,81) = 6.63, p = 0.014) for postural sway in the direction the stimulus (lateral sway). There was no significant main effect for ‘condition’ and there were no significant interactions between ‘condition’ and ‘visual motion’. The same analysis for anterior-posterior head movements in the response to the laterally moving stimulus showed no significant main effects or interactions. This shows that the postural responses were stimulus driven and appropriate for the visual signals.

**Figure 6:**
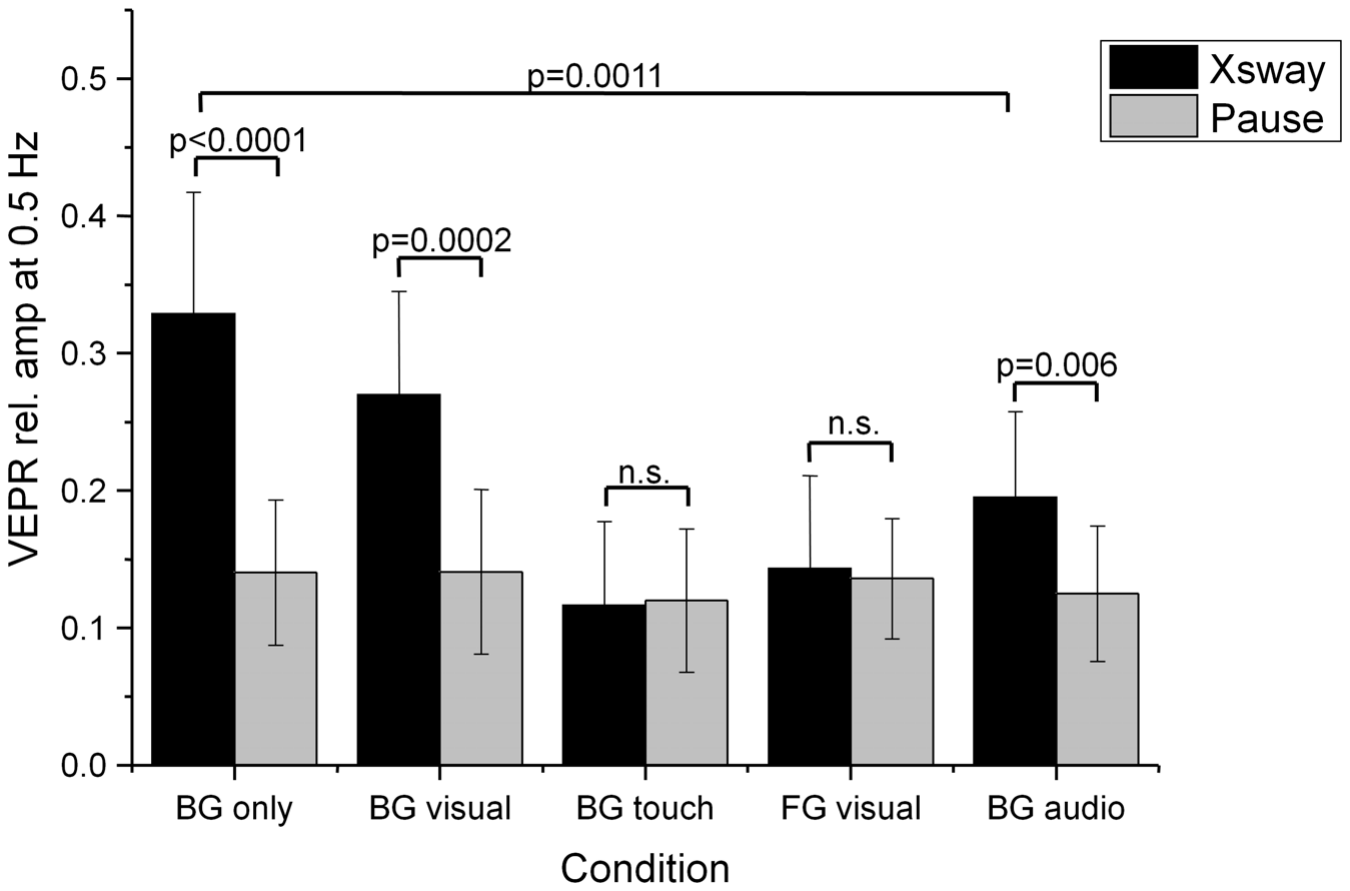
Relative amplitude of the postural sway response at the visual stimulus frequency (0.5 Hz) during visual stimulation (black bars) and without stimulus (Pause, grey bars). The labels identify the fixation point (BG/FG: background/foreground object fixation) and the type of anchor provided: visual – a rendered object in the foreground, touch – light finger touch, audio – a static white noise audio source.

The results of five planned comparisons of the visually evoked response with natural sway in the rest condition, summarised in table 1, show significant VEPRs when participants fixate on the moving background and no static foreground reference is present (condition ‘BG only’ in Fig. 6 and table 1). The VEPR has a peak amplitude of 0.9 mm and slightly lags behind visual stimulus, which is consistent with [18]. A slightly smaller (0.7 mm peak to peak), but highly significant, VEPR in the same phase is also seen where a visual reference is present, but not fixated (condition ‘BG visual’). Bronstein and Buckwell [18] report a cessation of consistent VEPRs in this condition but report a VEPR in the opposite direction to the visual stimulus when a foreground object is fixated (‘FG visual’), consistent with parallax-evoked automatic postural correction. No significant differences were found in the magnitude of the VEPR (14.3% of head motion energy at 0.5 Hz) compared to rest (13.0%, see table 1) in this condition.

**Table 1:** Lateral Sway energy at 0.5 Hz during visual stimulation and rest and comparison statistics for the target and control conditions in experiment 1a.

Condition	% Energy at 0.5 Hz Visual Motion	% Energy at 0.5 Hz Rest	Difference
**BG only**	**32.9% (s.d. = 8.8%)**	**14.0% (s.d. = 5.5%)**	**t_(18)_ = 5.76, p<0.0001**
**BG visual**	**27.0% (s.d. = 7.5%)**	**14.1% (s.d. = 6.0%)**	**t_(18)_ = 4.24, p = 0.0002**
BG touch	11.6% (s.d. = 6.1%)	11.9% (s.d. = 5.2%)	t**_(18)_** = 0.15, p = 0.4412
FG visual	14.3% (s.d. = 6.8%)	13.0% (s.d. = 4.4%)	t**_(18)_** = 0.28, p = 0.3913
**BG audio**	**19.5% (s.d. = 6.2%)**	**12.5% (s.d. = 4.9%)**	**t_(18)_ = 2.78, p = 0.0062**

Comparisons where there are significant differences between the visual stimulation and rest condition (Bonferroni corrected alpha levels of p = 0.01, (0.05/5)) are highlighted in bold.

Providing a static tactile reference (condition ‘BG touch’) leads to an elimination of VEPRs. When participants are asked to fixate on the background while a static white noise was played as an auditory reference (‘BG audio’, Fig. 6), significant VEPRs are evoked. A direct comparison with the equivalent condition (‘BG visual’, Fig. 6), however, shows a significant reduction of the relative amplitude of the stimulus driven frequency component (*t = 3.9, df = 18, p = 0.0011, two-sided*).

#### Experiment 1b: Postural responses to visual stimuli moving in the anterior-posterior plane

If fixation and configuration of the environment modulate VEPRs for lateral visual motion then similar effects might be expected for anterior-posterior motion. The aim of the second part of the experiment consequently was to measure VEPRs in response to front-back motion of the visual background.

**Figure 7:**
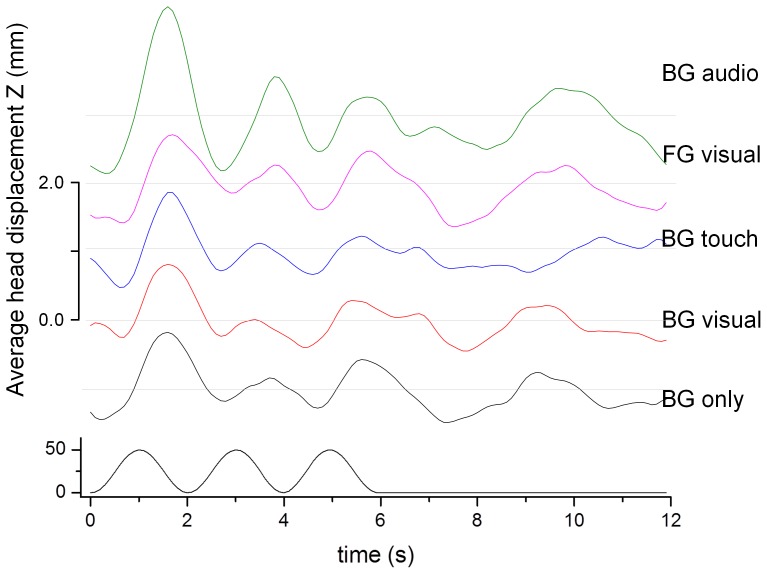
Average head displacement (top) in response to the anterior-posterior visual motion stimulus (bottom) in mm. The graphs are offset by 1.0 mm to enhance visibility.

Figure 7 shows the average head displacement in response to the visual background motion in the anterior-posterior direction. A consistent VEPR with a magnitude between 1 mm and 2.3 mm is clearly visible in all five experimental conditions.

An ANOVA with the factors ‘condition’, ‘visual motion’ and ‘subject’ as random factor showed significant main effects of ‘visual motion’ (F(1,81) = 37.21, p<0.0001) for postural sway in the direction the stimulus (anterior-posterior sway). There was no significant main effect for ‘condition’ and no significant interactions between ‘condition’ and ‘visual motion’. The same analysis for head sway in the lateral direction in response to the perpendicularly moving stimulus showed no significant main effects or interactions. This shows that the postural responses were stimulus driven and appropriate for the visual signals.

**Figure 8:**
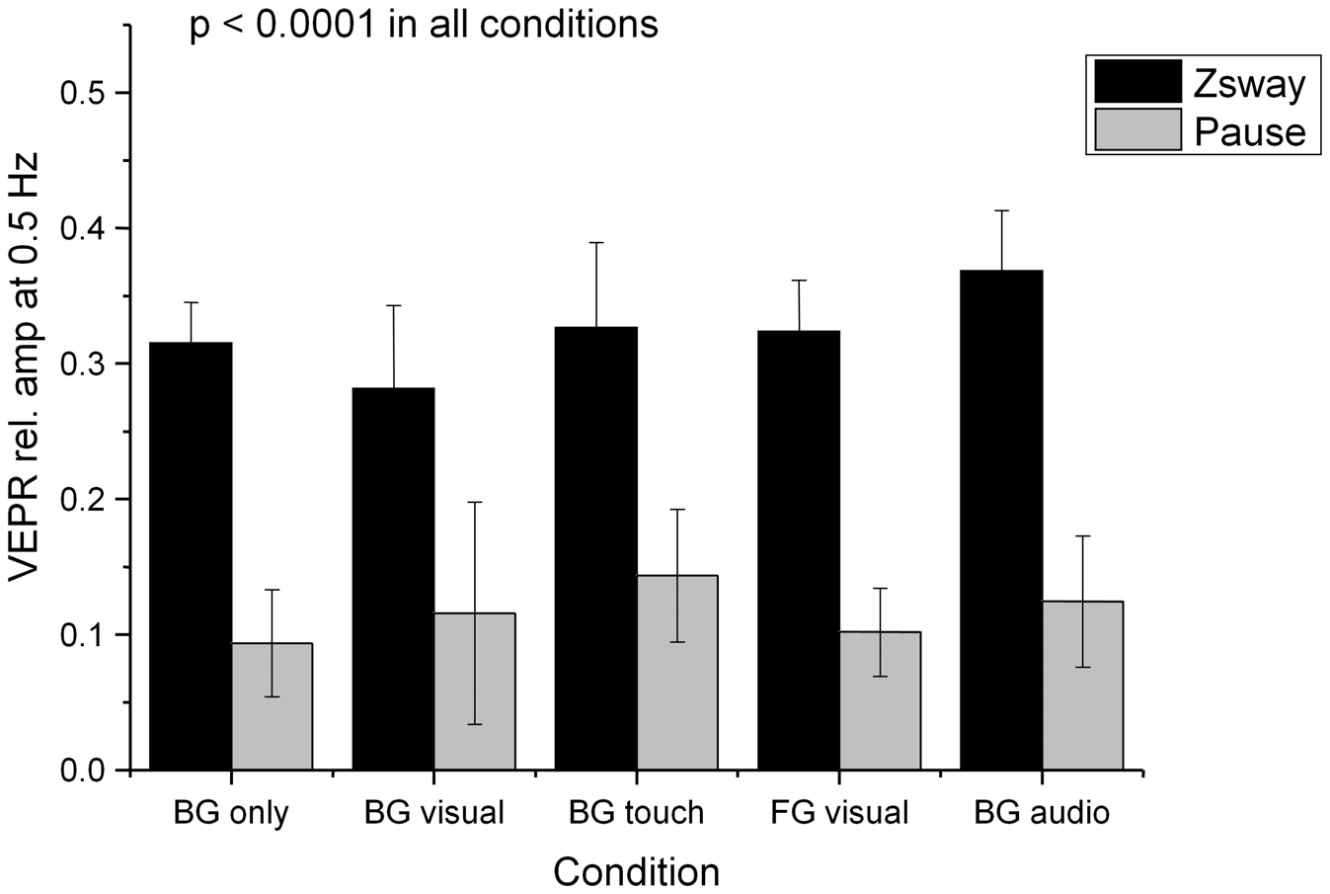
Relative amplitude of the postural sway response at the visual stimulus frequency (0.5 Hz) during visual stimulation (black bars) and without stimulus (Pause, grey bars) in the anterior-posterior plane. The labels identify the fixation point (BG/FG: background/foreground object fixation) and the type of anchor provided: visual – a rendered object in the foreground, touch – light finger touch, audio – a static white noise audio source.

Figure 8 and a summary of the VEPR data and statistical comparisons in table 2 shows highly significant (p<0.0001) visually evoked postural sway responses were observed in planned comparisons for all five experimental conditions compared to the control condition where no background motion was displayed.

**Table 2:** Anterior-posterior sway energy at 0.5 Hz during visual stimulation and rest and comparison statistics for the target and control conditions in experiment 1b.

Condition	% Energy at 0.5 Hz Visual Motion	% Energy at 0.5 Hz Rest	Difference
**BG only**	**31.5% (s.d. = 3.0%)**	**9.3% (s.d. = 3.9%)**	**t_(18)_ = 14.14, p<0.0001**
**BG visual**	**28.1% (s.d. = 6.1%)**	**11.6% (s.d. = 8.2%)**	**t_(18)_ = 5.10, p<0.0001**
**BG touch**	**32.6% (s.d. = 6.3%)**	**14.4% (s.d. = 4.9%)**	**t_(18)_ = 7.27, p<0.0001**
**FG visual**	**32.4% (s.d. = 3.7%)**	**10.2% (s.d. = 3.3%)**	**t_(18)_ = 14.24, p<0.0001**
**BG audio**	**36.9% (s.d. = 4.4%)**	**12.4% (s.d. = 4.9% )**	**t_(18)_ = 11.79, p<0.0001**

All comparisons show significant differences between the visual stimulation and rest condition (Bonferroni corrected alpha levels of p = 0.01, (0.05/5)).

#### Summary

The three visual conditions (BG only, BS visual, and FG visual) in experiment 1a were designed to replicate data from Bronstein and Buckwell [18]. While the observed postural response in the ‘BG only’ condition matches previously reported data for lateral motion, neither of the other two conditions (BG visual and FG visual, Fig. 2) are consistent with it.

It was expected that fixating the background in the presence of a foreground object would eliminate postural responses, while the observation was a VEPR of comparable magnitude and phase as in the ‘BG only’ condition. A reversal of postural sway in the fixate foreground (FG visual) condition was expected, but no consistent postural response was observed in this condition.

When visual motion is presented as anterior-posterior motion rather than lateral motion, consistent visually evoked postural responses are seen that are unaffected by the configuration of the visual environment or tactile or auditory references.

### Experiment 2: The Effect of Static Real and Virtual Visual Reference Signals on Visually Evoked Postural Responses

The observed behaviour in experiment 1a differed significantly from previously reported data [18], [26]. To test whether the presentation of the virtual foreground object affected the postural response a second experiment was conducted, comparing VEPRs in matching conditions where the visual reference was either a physical object or a virtual representation. The spatial position of the real and virtual foreground object was matched. The ‘virtual’ conditions were the same as in experiment 1 to validate the original results, in the ‘real’ conditions a physical teapot, on a stand and in the same position as its virtual counterpart, was used.

Each experiment consisted of five conditions, labelled as follows:

1) BG only - fixate a target on the background (BG), no foreground objects present.

2) BG virtual - fixate a target on the background, a virtual foreground object is present.

3) BG real - fixate a target on the background, a real foreground object is present.

4) FG virtual - fixate a target on a virtual rendering of the foreground (FG) object.

5) FG real - fixate a target on a real foreground object.

Conditions 1, 3 and 5 are similar to those used in [18], conditions 1,2 and 4 replicate results from experiment 1.

#### Behavioural performance

A separate group of ten participants took part in these experiments.

To ensure that participants engaged in the experiment and fixated the target marker, they were asked to respond by pressing a button when the target transiently disappeared. Participants correctly identified 99.25% (s.d. 1.27%) of these events in the lateral sway condition and 98.81% (s.d. 1.36%) in the anterior-posterior motion condition. All participants responded correctly to at least 95% of trials.

#### Experiment 2a: postural responses to visual stimuli moving in the lateral plane

A repeated measures ANOVA with the factors ‘condition’, ‘visual motion’ and ‘subject’ as a random factor showed significant main effects of visual motion (*F(1,81) = 9.84, p = 0.002*) and significant interactions between condition and visual motion (*F(4,81) = 2.75, p = 0.033*) for postural sway in the lateral direction. The same analysis for anterior-posterior sway showed no significant main effects or interactions as would be expected for stimulus driven behaviour.

**Figure 9:**
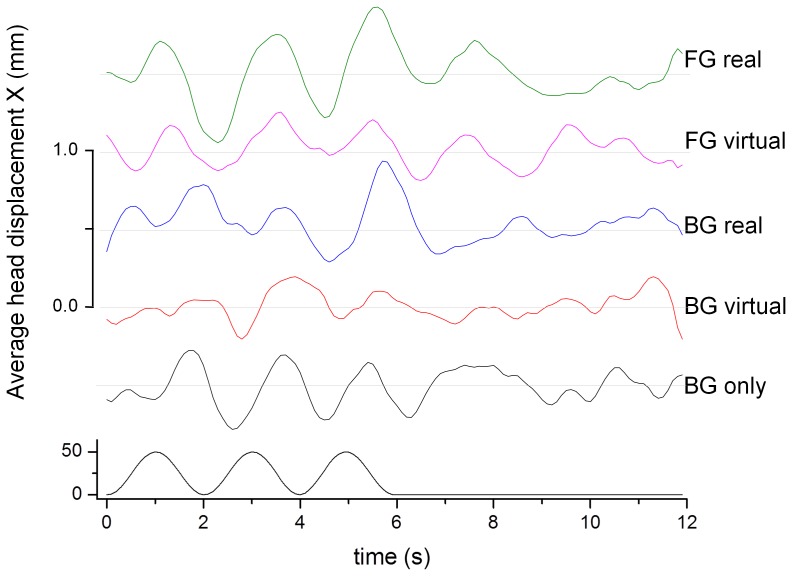
Average head displacement (top) in response to the laterally moving visual stimulus (bottom) in mm. The graphs are offset by 0.5 mm to enhance visibility. The labels identify the fixation point (BG/FG: background/foreground object fixation) and whether the foreground object was a virtual representation or a physical object (real).

**Figure 10:**
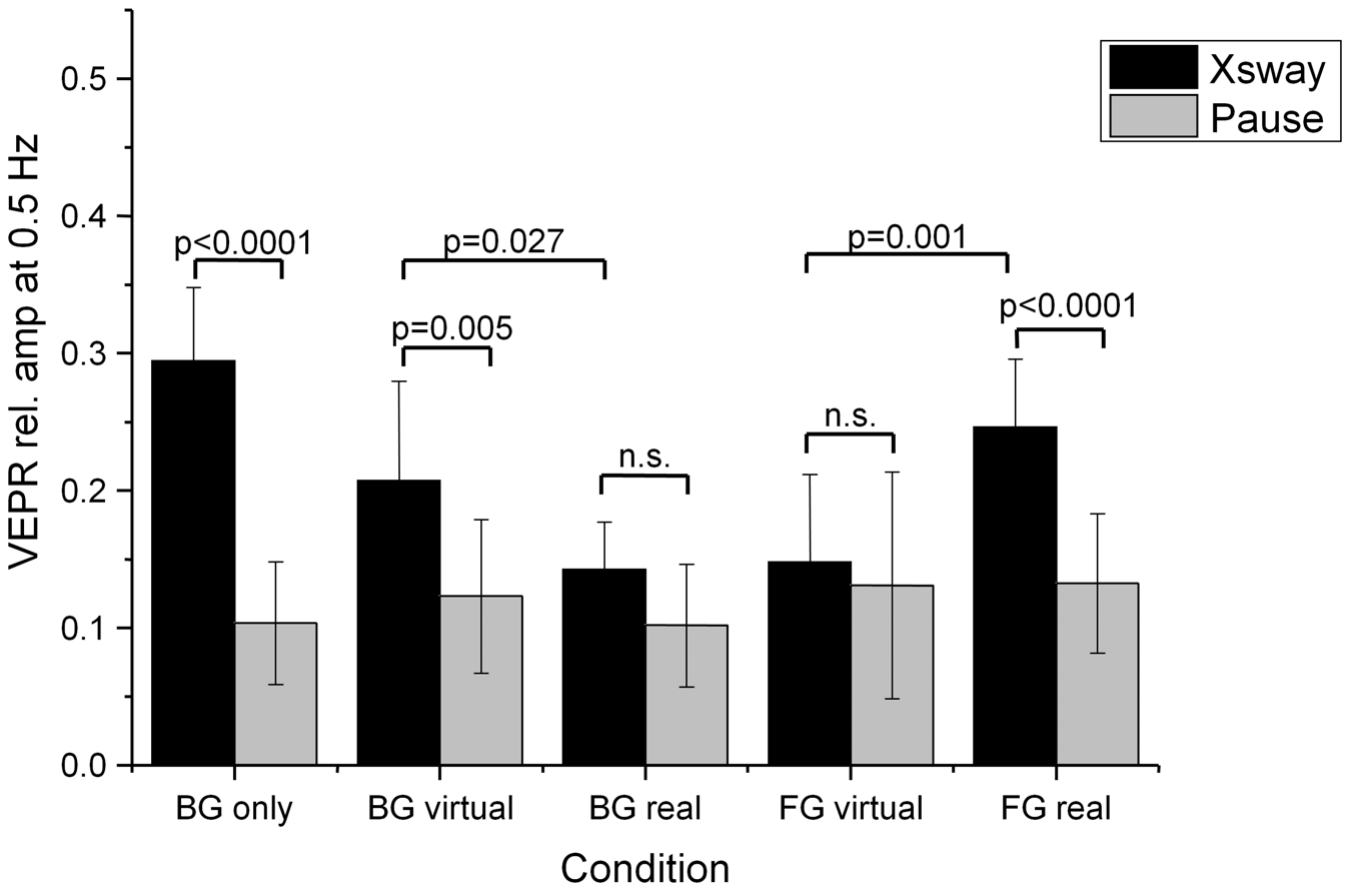
Relative amplitude of the postural sway response at the visual stimulus frequency (0.5 Hz) during lateral motion of the background (black bars) and without background motion (Pause, grey bars). The labels identify the fixation point (BG/FG: background/foreground object fixation) and whether the foreground object was a virtual representation or a physical object (real). Conditions ‘BG virtual’/‘BG real’ and ‘FG virtual’/‘FG real’ represent equivalent stimulus configurations where responses to real and virtually represented objects are compared.

The mean head displacement data for the five conditions is shown in figure 9. An analysis of the VEPR components at 0.5 Hz and comparison with the same frequency component when no visual motion is present (Pause, Fig. 10) shows that postural responses for real foreground objects differ from VEPRs when virtual foreground objects are present in the same position.

The summary data in table 3 shows that background fixation without a foreground object being present causes significant VEPRs (condition ‘BG only’). The magnitude of the response (0.5 mm peak to peak) and, critically the relative magnitude at the VEPR at 0.5 Hz (29.5%) are comparable with the same condition in experiment 1a (amplitude 0.7 mm peak to peak, relative VEPR magnitude 31.5%). As in experiment 1a a significant VEPR is measured when the background is fixated in the presence of a virtual foreground object (in condition ‘BG virtual’ or ‘BG visual’ in experiment 1a). The relative VEPR magnitudes are also comparable: 26.7% in experiment 2a vs. 28.1% in experiment 1a.

**Table 3:** Sway energy at 0.5 Hz during lateral visual stimulation and rest and comparison statistics for the target and control conditions in experiment 2a.

Condition	% Energy at 0.5 Hz Visual Motion	% Energy at 0.5 Hz Rest	Difference
**BG only**	**29.5% (s.d. = 5.3%)**	**10.4% (s.d. = 4.5%)**	**t_(18)_ = 8.71, p<0.0001**
**BG virtual**	**26.7% (s.d. = 7.2%)**	**14.3% (s.d. = 5.6%)**	**t _(18)_ = 2.91, p = 0.0047**
BG real	14.3% (s.d. = 5.6%)	10.2% (s.d. = 4.4%)	t **_(18)_** = 1.79, p = 0.0451
FG virtual	14.8% (s.d. = 6.4%)	13.1% (s.d. = 8.3%)	t **_(18)_** = 0.51, p = 0.3081
**FG real**	**24.6% (s.d. = 5.0%)**	**13.2% (s.d. = 5.1%)**	**t _(18)_ = 5.08, p<0.0001**

Comparisons where there are significant differences between the visual stimulation and rest condition (Bonferroni corrected alpha levels of p = 0.0071, (0.05/7)) are highlighted in bold.

Maintaining fixation at the background, but replacing the virtual foreground object with a real teapot in the same position caused a cessation of significant VEPRs components in the direction and at the frequency of the visual stimulus (table 3, BG real). The experimental setup, and participant behaviour in the ‘BG real’ condition mirrors the data reported in [18].

A direct comparison of the visually evoked sway in the two conditions shows significantly more postural sway in the ‘BG virtual’ than in the ‘BG real’ condition (*t_18_ = 2.4052, p = 0.0027, two-sided*).

A difference in postural response is also seen when participants fixate the real and virtual foreground objects. As in experiment 1a, no significant VEPR is seen when the second cohort of participants fixate on a virtual foreground object (‘FG virtual’, table 3). Fixating on a matching real foreground object (‘FG real’), however leads to significant postural responses.

A direct comparing of the two visually driven conditions with real and virtual fixated foreground objects reveals a significant difference in postural sway (*t_18_ = 3.8294 p = 0.0012, two-sided*). These data, again confirm the results for the virtual presentation in experiment 1 and previously reported data with physical static foreground objects [18].

**Figure 11:**
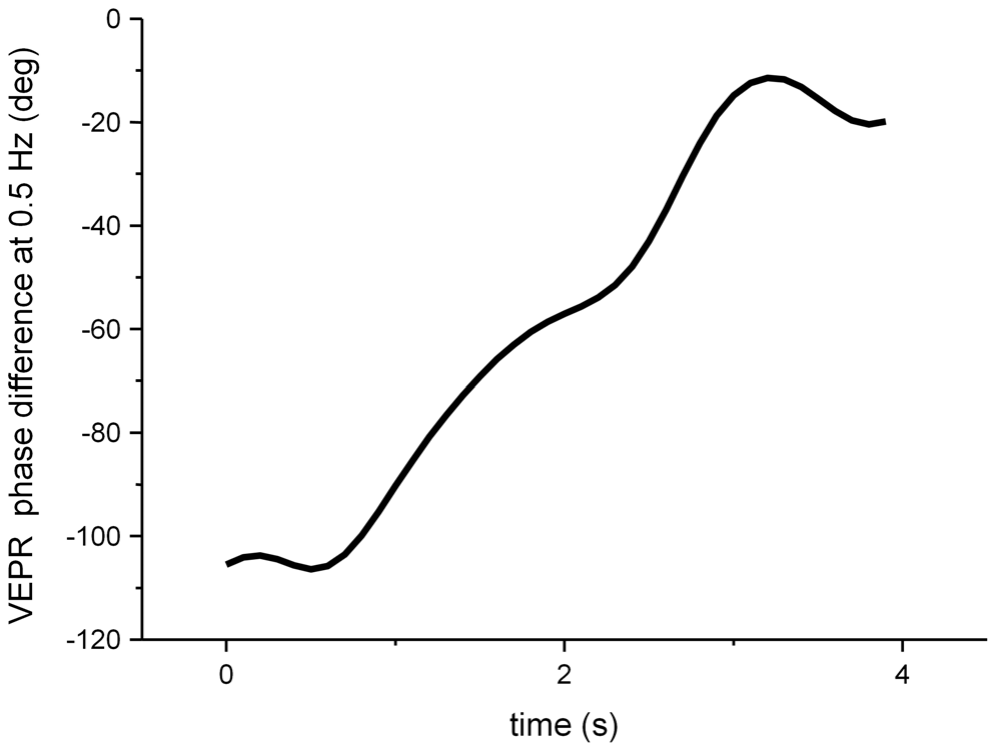
Difference in VEPR phase at 0.5 Hz between ‘FG real’ and ‘BG real’ conditions, computed in a moving analysis window (2 s Hanning) starting at the times shown. The graph shows that initially the VEPR for the BG real response lags 110° behind the FG real VEPR but as the analysis window moves through the VEPR, the phase difference reduces to approximately 20°.

It has previously been reported that fixating a static foreground object reverses the direction of the VEPR compared to that observed when only the background is present and fixated. Figure 11 shows an analysis of the phase relationship between the two responses. The phase difference between the two population responses was computed using a moving Hanning window of 2 s duration that started between 0 s and 4 s relative to the visual stimulus onset. The data shows a gradual shift from −110° at the stimulus onset to −20° at the ends of the visual stimulus (4–6s).

Our experimental data in the virtual conditions replicates the behaviour seen in the equivalent conditions of experiment 1a, and differ significantly from the behaviour observed when the virtual foreground object is replaced with a real object in the same position.

In conditions where a real foreground object is present, participant’s behaviour is similar to that described by Bronstein and Buckwell [18]. When participants fixate the background the evoked sway is not significantly different from natural postural sway in the rest condition (condition BG real). Fixating a real foreground object, however, leads to significant evoked postural sway.

Our data replicates the original experiments [18], [27] that showed a counter-directional response for transient (2s) visual stimuli. We observe an initial partial phase inversion (−110°), which then dissipates over the first four seconds of stimulus presentation. This phase lag is consistent with data reported in [18].

#### Experiment 2b – Anterior-posterior sway with real and virtual foreground objects

The second part of experiment 2 replicates the conditions used experiment 2a but, as in experiment 1b, uses anterior-posterior rather than lateral motion as a visual stimulus.

**Figure 12:**
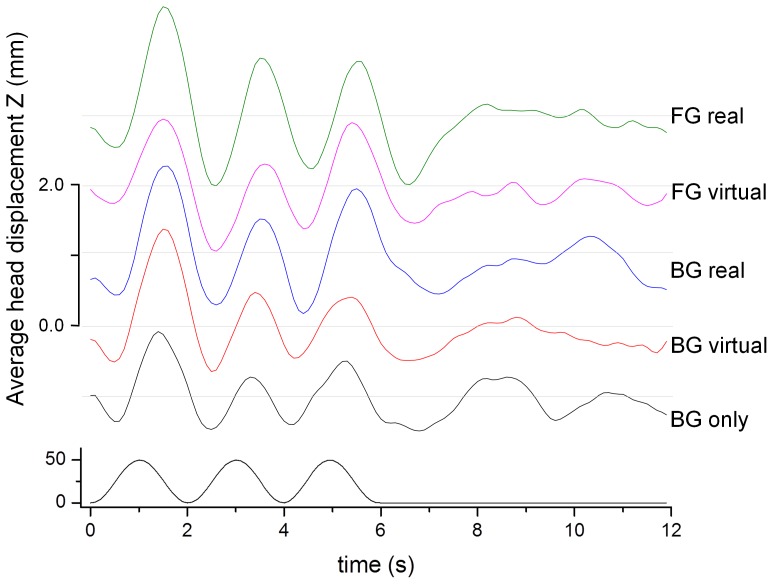
Average head displacement (top) in response to the anterior-posterior visual motion stimulus (bottom) in mm. The graphs are offset by 1.0 mm to enhance visibility.

We observe consistent head displacement data that is clearly locked into the visual stimulus in all experimental conditions (Fig. 12). The peak-to-peak amplitude of the mean head displacement varied between 1.5 mm (BG only) and 2.5 mm (FG real).

**Figure 13:**
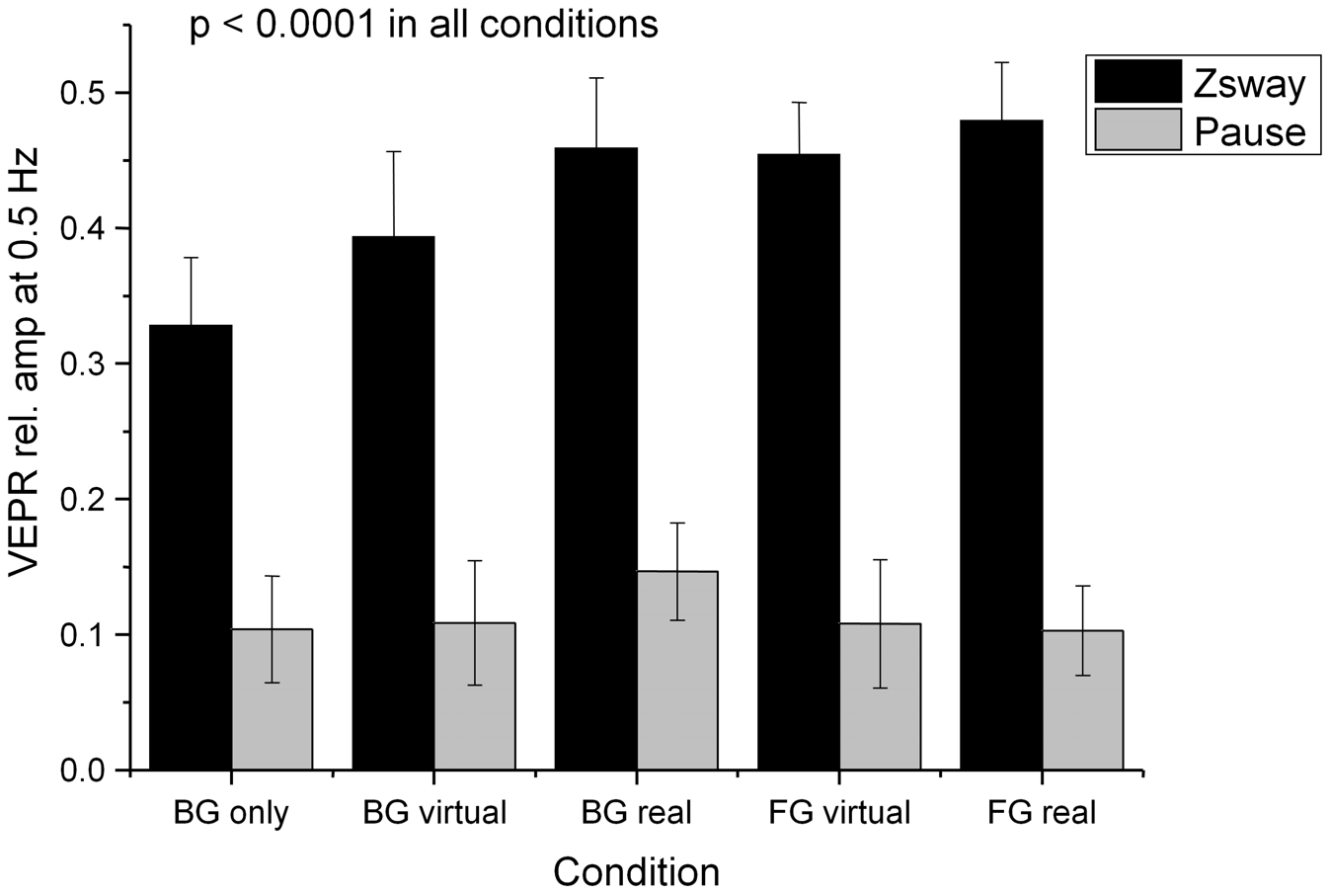
Postural sway in Z direction during visual stimulation (back bars) and pause (grey bars) in the anterior-posterior direction. The conditions are the same as shown in Fig. 10.

The relative VEPR components at 0.5 Hz during visual stimulation and for the rest condition that immediately followed each stimulus type are shown in Fig. 13. A repeated measures ANOVA with the factors condition, visual motion (on, pause) and subject as random factor showed significant main effects of visual motion (F(1,81) = 121.95, p<0.0001) but no other main effects or interactions for postural sway in anterior-posterior direction. Postural sway in the lateral direction did not differ significantly between visual stimulation and rest (F(1,81) = 2.67, p = 0.11) when the visual signal moved in the perpendicular direction.

We observe significant (p<0.0001) differences in the amplitude of stimulus-evoked body sway at 0.5 Hz compared to the rest condition between trials in *all* experimental conditions, table 4.

**Table 4:** Sway energy at 0.5 Hz during visual stimulation and rest and comparison statistics for the target and control condition in experiment 2b.

Condition	% Energy at 0.5 Hz Visual Motion	% Energy at 0.5 Hz Rest	Difference
**BG only**	**32.7% (s.d. = 5.07%)**	**9.7% (s.d. = 3.91%)**	**t_(18)_ = 11.37, p<0.0001**
**BG virtual**	**39.6% (s.d. = 6.02%)**	**11.1% (s.d. = 4.66%)**	**t_(18)_ = 11.83, p<0.0001**
**BG real**	**46.1% (s.d. = 5.05%)**	**14.8% (s.d. = 3.64%)**	**t_(18)_ = 15.97, p<0.0001**
**FG virtual**	**45.3% (s.d. = 4.06%)**	**10.8% (s.d. = 4.8%)**	**t_(18)_ = 16.32, p<0.0001**
**FG real**	**47.8% (s.d. = 4.65%)**	**10.5% (s.d. = 3.42%)**	**t_(18)_ = 20.51, p<0.0001**

All comparisons showed significant differences between the visual stimulation and rest condition (Bonferroni corrected alpha levels of p = 0.0071, (0.05/7)).

The results of this experiment mirror these of experiment 1b where it was shown that background motion in the anterior-posterior plane evokes consistently strong postural sway. The fixation point or nature of the foreground object in the scene does not affect the observed responses.

### Conclusion

The primary aim of this paper was to explore how external visual, haptic and auditory positional references modulate automatic visually evoked postural sway. We show that tactile and auditory static reference signals in virtual environments effectively reduce VEPRs to laterally moving stimuli but do not affect the response to visual motion stimuli that move in the anterior-posterior plane.

Experiment one was designed to replicate and extend previous work showing a differential response of VEPRs depending on the fixation point and the presence of static foreground objects. Postural sway in line with the background motion was expected and observed when no foreground reference objects are present. Although a state-of-the art virtual environment, with large, high resolution 3D display, and high resolution observer motion tracking, was used, it was found that the VEPRs for lateral motion differed from those predicted by [18], [26]. A second experiment, where participants were presented with real and virtual reference objects in the same position, confirmed the results of first experiment and shows different VEPRs for real and virtual reference objects. When real objects were positioned in the foreground, our participant’s behaviour matched previously reported data for similar experimental conditions [18], [26].

The experiments show that VEPRs to laterally moving visual stimuli are modulated by the fixation point and stimulus configuration. Importantly, they also showed qualitatively and quantitatively different behaviour for real and virtually presented foreground objects.

Since the transient VEPRs observed in this study are considered subconscious and automatic responses to visual stimuli [26] we propose the experimental framework as a critical test for the fidelity of VR systems. The context dependent postural adaptation to laterally moving visual stimuli should be the same for real and virtual foreground signals if observers perceive both environments as equally real or are equally present in them. The stimuli used are very simple and the experiments do not require equipment beyond the visual presentation and head tracking devices that are integral parts of most modern VR environments. Robust responses were measured for a relatively small group of participants so that a ‘postural response test’ could be part of the verification or commissioning of new or existing VR environments.

Significant differences were observed in responses to lateral and anterior-posterior visual motion. VEPRs to lateral motion are modulated by the environment while VEPRs in the anterior-posterior visual motion direction are surprisingly robust: even touching a static reference point with a finger did not reduce the anterior-posterior VEPR in experiment 1b.

Anterior-posterior VEPRs are unaffected by visual, tactile and audio cues that eliminate or significantly reduce postural sway for lateral motion of equal magnitude.

## Discussion

### Differences in Lateral and Anterior-posterior Sway

One of the most striking differences lies in the postural response patterns to lateral and anterior-posterior motion. Lateral motion is modulated by the presence and nature of static reference objects in the environment while visual motion signals in the anterior-posterior direction cause highly significant posture adjustments independently of the fixation point or the presence of static reference points.

The nature of the motion signal, in our view, offers an explanation why the fixation point does not affect the response: anterior-posterior motion is signalled by an expansion of the retinal image. In real life, and in VR systems that adjusts the view by tracking the observer position, this motion signal is minimal directly ahead of the observer and maximal in the most peripheral areas of the view.

An inversion of the postural response analogous to that induced by the lateral (parallax) motion condition was not expected, because any postural control system using parallax cues should not respond to looming or receding motion signals. A static foreground anchor was expected to, at least, reduce postural sway. The experimental results provide no evidence for this: neither fixating a real foreground object that provides veridical position information, nor using finger touch as an anchor affects the amplitude or phase of the VEPR.

In terms of VR systems design this means that this motion component, since it is much harder to disrupt than lateral visual motion, may provide a useful mechanism to increase immersion. Large scale anterior-posterior background motion, conversely, should be avoided in applications where immersion, vection, or postural sway are not desired features, such as in collaborative work environments and data visualisation applications. This finding is consistent with the arguments made by Riecke [3], [5].

### The Effect of Visual, Auditory and Tactile Anchors on Postural Sway

Real and virtual objects in the environment differentially modulate the VEPR for laterally moving visual backgrounds. The introduction of a real object that provides a visual anchor while participants fixate a moving background abolishes VEPRs that are seen when only the background is present. A virtual representation of the same object does not have an effect on the VEPR. The provision of a tactile anchor during background fixation extincts VEPRs in our environment, while the provision of an auditory static spatial reference significantly reduces postural sway.

This shows that all these cues are effective in modulating immersion in virtual environments. To maximise immersion, cues that are incongruent with the intended motion patterns should be minimised. We note that a static white noise source significantly reduces VEPRs in experiment 1b. Most VR environments use noisy computers and projection systems that may provide cues, similar to the signals we used. Deliberate steps to reduce external noise or the introduction of targeted, moving audio cues are likely to improve immersion and fidelity in VR settings [5], [51]. It is technically difficult to provide dynamic tactile signals in VR environments so that it may be difficult to exploit tactile cues to increase presence. Tactile cues, however, are a relatively simple way to reduce immersion where necessary.

Touching a stationary surface with a finger abolished consistent visually driven postural sway in the lateral visual motion condition, (condition BG touch, Fig. 6) but not in the anterior-posterior condition (Fig. 8). Lishman and Lee [52] reported that vection influences posture during stationary stance even when visual information conflicts with other sensory information. They concluded that vision is an autonomous kinaesthetic sense that operates independently of mechanical (articular, cutaneous, and vestibular) kinaesthesis. Our data support this hypothesis for the anterior-posterior visual motion signals but the responses for the lateral motion show that tactile information influences VEPRs for lateral visual motion.

### Contra-directional Responses when Real Foreground Objects Provide Parallax Motion Cues

Earlier studies [18], [26] that showed clear evidence for postural responses in the opposite direction to that observed in the condition where only the background was present, we observed a 110° phase shift in the evoked response during for the initial two seconds of visual stimulus presence. After this period the phase response gradually changed and matched that seen for the ‘BG only’ condition after four seconds. The original study [18] used a single cycle of background motion and did not quantify the phase of the postural response, but an inspection of their data shows that the postural response was inverted and slightly delayed, consistent with the initial 110° phase shift observed in experiment 2a.

A possible explanation for this behaviour may be provided by Guerraz and Bronstein [26] who argue that two separate systems interact in VEPR generation: a transient, automatic system that takes parallax motion into account and a much slower system that doesn’t. Earlier work shows that parallax evoked postural sway of modulated by the fixation point, which suggests that foveal signals dominate processing [18], while the slower pathway proposed by [26], which leads to vection, is likely to draw on peripheral vision to provide the relevant motion cues [15], [53]. An important difference between the VR experiments we describe and those reported earlier is the size of the display and with it the relative contribution of foveal and peripheral vision to overall postural processing. We speculate that, just like fixation, the relative availability and saliency of foveal and peripheral visual cues is likely to modulate the VEPR in conditions where a static foreground object is present.

### Postural Sway as an Indicator of Presence and Fidelity in Virtual Reality

Visually evoked postural responses are one manifestation of a system that integrates postural and visual signals to maintain balance but that also forms the basis of perceived vection and presence in virtual reality. The results show that the system not only integrates visual and directly relevant somatosensory information but also auditory and not directly posturally relevant finger touch. They replicate findings that show that the initial component of postural sway is modulated by the geometry of the space but also by the fixation point. This shows that fine detail, which requires sophisticated cortical representations, such as the relative amounts of foreground and background motion, contribute to postural responses. We hypothesize that the fidelity of the virtual environment and a concurrent evaluation of the plausibility or positional stability of the reference points present in the scene also contributes to the postural response.

A user’s subjective psychological response to a VR system, the feeling of being within the simulated environment rather than watching it from the outside, is termed *presence* [2]. Inducing this sense of presence is an important aim in the design of virtual reality systems because increasing presence and fidelity will improve immersion and consequently task performance [4], [5], [54]. Presence, however, is not a single construct, but depends on a number of factors, such as the degree of immersion in and interaction with the virtual environment [55]. Schubert et al. [56], for example, argue that presence results from an interpretation of a mental model of the virtual environment that draws on two primary independent components: spatial-constructive features and attention, while simulation fidelity provides a further subjective component. Consistent and discriminative subjective ratings of fidelity and presence of virtual reality systems are difficult to obtain, in particular using moderately sized pools of participants, and can be biased by previous judgments of the same stimuli [51], [57].

Behavioural responses as objective measures of fidelity and presence offer a potential alternative to subjective ratings. Postural response differences would be a useful objective measure of spatial presence in the virtual environment because context and fixation dependent differential responses can be estimated robustly with relatively small participant pools. The VEPRs are a direct and unconscious adaptation to changes in the perceived spatial environment and are modulated by visual attention. The postural responses can be elicited without specific attention to the task.

We argue that the automatic postural adaptation in response to changes in the mediated virtual environment is a potential measure of presence because it depends on the degree to which visual stimuli in virtual environments are perceived as self-motion, in other words as a measure to what extent the observer feels part of, and present in, the virtual environment.

Future work should evaluate which factors modulate postural VEPRs: examples include the fidelity of rendering, the precision and time lag of motion tracking, prior expectations of participants about foreground object behaviour and stability, the participant’s task, cognitive load, or even anomalies on self-awareness, such as depersonalisation disorder. A formal evaluation of the link between objective (postural response magnitude) and subjective (questionnaire based [55]) measures of presence would provide the basis for the systematic application of the test for an evaluation of VR systems.

### Controlling Vection and Presence in Virtual Environments

The argument that automatic postural reactions to vection are a useful measure of presence in mediated environments may be taken to imply that systems should be designed to maximise vection. The reality is more complex. This project was conceived to address the unpleasant aspects of vection that are commonly seen in large scale collaborative VR environments where one participant controls the (shared) view of a number of participants in a simulation. Movements that result in viewpoint changes and also the manipulation of large 3D objects that form the centre of attention should be perceived as object motion, not self-motion. Our results show that postural responses to lateral motion can be minimised by providing either attended virtual or unattended physical objects within the participants view or providing auditory, visual or tactile anchor points. Anterior-posterior visual motion, which is predominantly mediated by peripheral visual cues, is not affected by these anchors, but can be reduced by restricting the field of view within a simulation. The deliberate avoidance of non-information bearing signals, such as backgrounds will also reduce vection.

Conversely, postural responses, vection, and presumably presence, can be maximised by providing naturalistic environments where particular care is taken to provide a naturalistic visual background and to avoid visual, auditory and haptic anchors. Riecke [5] argues that manipulating the perceived object-background separation might be the most effective means of achieving this aim, for collaborative VR environments, this implies that individual views should be presented for all participants of a simulation, which requires separate or head mounted displays and individual motion tracking.
